# OxLDL as an Inducer of a Metabolic Shift in Cancer Cells

**DOI:** 10.7150/jca.56307

**Published:** 2021-08-03

**Authors:** Albert V. Bitorina, Yvonne Oligschlaeger, Lingling Ding, Tulasi Yadati, Annemarie Westheim, Tom Houben, Rianne D.W. Vaes, Steven W.M. Olde Damink, Jan Theys, Ronit Shiri-Sverdlov

**Affiliations:** 1 Department of Molecular Genetics, School for Nutrition and Translational Research in Metabolism (NUTRIM), Maastricht University, Maastricht, The Netherlands.; 2 Department of Surgery, School for Nutrition and Translational Research in Metabolism (NUTRIM), Maastricht University, Maastricht, The Netherlands.; 3 Department of Precision Medicine, School for Oncology & Developmental Biology (GROW), Maastricht University Medical Centre, Maastricht, Netherlands.

**Keywords:** Pancreatic cancer cells, oxLDL, metabolic switch, HIF-1α, autophagy

## Abstract

Recent evidence established a link between disturbed lipid metabolism and increased risk for cancer. One of the most prominent features related to disturbed lipid metabolism is an increased production of oxidized low-density-lipoproteins (oxLDL), which results from elevated oxidative stress. OxLDL is known to have detrimental effects on healthy cells and plays a primary role in diseases related to the metabolic syndrome. Nevertheless, so far, the exact role of oxLDL in cancer cell metabolism is not yet known. To examine changes in metabolic profile induced by oxLDL, pancreatic KLM-1 cells were treated with oxLDL in a concentration- (25 or 50 µg/ml) and/or time-dependent (4 hr or 8 hr) manner and the impact of oxLDL on oxygen consumption rates (OCR) as well as extracellular acidification rates (ECAR) was analyzed using Seahorse technology. Subsequently, to establish the link between oxLDL and glycolysis, stabilization of the master regulator hypoxia-inducible factor 1-alpha (HIF-1α) was measured by means of Western blot. Furthermore, autophagic responses were assessed by measuring protein levels of the autophagosomal marker LC3B-II. Finally, the therapeutic potential of natural anti-oxLDL IgM antibodies in reversing these effects was tested. Incubation of KLM-1 cells with oxLDL shifted the energy balance towards a more glycolytic phenotype, which is an important hallmark of cancer cells. These data were supported by measurement of increased oxLDL-mediated HIF-1α stabilization. In line, oxLDL incubation also increased the levels of LC3B-II, suggesting an elevated autophagic response. Importantly, antibodies against oxLDL were able to reverse these oxLDL-mediated metabolic effects. Our data provides a novel proof-of-concept that oxLDL induces a shift in energy balance. These data not only support a role for oxLDL in the progression of cancer but also suggest the possibility of targeting oxLDL as a therapeutic option in cancer.

## Introduction

Cancer is the second leading cause of deaths worldwide, which currently accounts for nearly ten million deaths 1. In particular, overweight and obesity, due to excess energy intake and decreased physical activity, have reached pandemic levels worldwide 2 and recent clinical data suggests that individuals with obesity are at an increased risk of developing several types of cancer 3-5.

Mechanisms that are known to link obesity with increased cancer burden, *i.e.*, cancer development, progression and decreased treatment response 6, include adipose tissue dysfunction, increased circulating levels of hormones, perturbed lipid metabolism and low-grade chronic inflammation 7. Chronic inflammation is strongly associated with increased secretion of cytokines in the circulation, which can lead to cancer-related cellular processes such as cell adhesion, chemotaxis, migration and angiogenesis 8. Moreover, excess nutrient intake has been shown to increase the expression of multiple growth factors and hormones 9, 10 and is known to induce changes in gut microbiota 11, thereby influencing oncogenesis and tumor progression.

One of the most prominent features related to excess nutrient intake and dysregulated lipid metabolism is the increased production of oxidized cholesterol-rich low-density lipoprotein (oxLDL), which results from elevated oxidative stress. Increased levels of oxLDL is known to have detrimental effects on healthy cells and plays a primary responsible role in metabolic syndrome (MetS)-related diseases such as atherosclerosis 12 and non-alcoholic steatohepatitis (NASH) 13. Strikingly, increasing evidence also points towards a correlation between circulating levels of oxLDL and the development of cancer 14, suggesting that oxLDL is a potent pro-oncogenic factor. Nevertheless, the exact metabolic role of oxLDL in cancer is not well-understood.

In the present study, we hypothesized that oxLDL induces a metabolic shift in cancer cells. We demonstrate for the first time that incubating KLM-1 pancreatic cancer cells with oxLDL shifts the energy balance towards a more glycolytic phenotype, which is supported by oxLDL-induced stabilization of hypoxia-inducible factor 1-alpha (HIF-1α), a master regulator of glycolysis. In line with these data, we also show that oxLDL, likely due to increased autophagic activity, increases the expression of the autophagosomal marker LC3B-II. Essentially, the addition of EO6 IgM antibodies against oxLDL were able to reverse the oxLDL-mediated metabolic effects.

Altogether, our data provide a proof-of-concept that oxLDL induces a metabolic shift, which is involved in cancer progression. In addition, our data also supports the opportunity of targeting oxLDL as a novel therapeutic option in cancer.

## Materials and methods

### Cell culture

Human pancreatic cancer cell line KLM-1 (obtained from RIKEN BioResource Center) was cultured in high glucose (4.5 g/L) Dulbecco's Modified Eagle's Medium (DMEM), supplemented with 10% (v/v) fetal bovine serum (USDA Approved, PAA, Pasching, Austria). Cells were maintained at 37 °C, 5% CO_2_ in a humidified incubator.

### Energy metabolism assay

KLM-1 cells were grown in DMEM + 10% FBS and seeded into 96XF plates @ 24000 cells/well and left to attach overnight at 37 °C. Metabolic profiles were generated by replacing the growth medium with assay medium 1 hr before measurement according to the manufacturer guidelines 15. Minimally oxidized LDL (Alfa Aesar) concentration was increased to 25 µg/ml over 60 min in steps of 5 µg/ml. Oxygen consumption and extracellular acidification rates were monitored using a Seahorse Bioscience XF96 Extracellular Flux Analyzer. Values were corrected *versus* baseline as well as for total protein content in each well. Total protein concentration was measured using a Pierce^®^ BCA protein assay kit (Waltham, MA, USA). Data were obtained and depicted using Wave 2.6.0.31 software (Agilent).

### Western blot

In 3 independent experiments, KLM-1 cells were grown in DMEM + 10% FBS and seeded into 6 well plates at 500,000 cells/well and left to attach overnight at 37 ºC. Subsequently, cells were treated with oxLDL (25 or 50 µg/ml; minimally oxidized; Tebu-Bio) for 4 hr or 8 hr. Alternatively, cells were either treated with oxLDL alone (50 µg/ml), oxLDL (50 µg/ml) + chloroquine (20 µM), oxLDL (50 µg/ml) + EO6 (14 µg/ml; Avanti) or oxLDL (50 µg/ml) + chloroquine (20 µM) + EO6 (14 µg/ml). Subsequently, cells were washed with cold PBS and lysed with cold RIPA buffer (50 mM Tris-HCl pH 7.5, 150 mM NaCl, 0.5% Sodium deoxycholate, 1% Triton X-100, 0.1% SDS) supplemented with a mixture of protease and phosphatase inhibitors (Complete and PhosStop; Roche). Lysates were centrifuged at 16,000 xg for 15 min, after which the supernatant was transferred to a new Eppendorf and stored at -20 °C. The total protein concentration was measured using a Pierce^®^ (Waltham, MA, USA) BCA protein assay. Equal amounts of protein were loaded on the gel. After SDS/PAGE, proteins were transferred to nitrocellulose membrane (Bio-Rad), which was blocked with 5% non-fat dry milk for 1h at room temperature. For detection, the membrane was incubated with an antibody against HIF-1α (1:2000 dilution; BD Transduction Laboratories; Catalogue #610959), β-Actin (1:2000 dilution; Cell Signaling, #4967) and LC3B (1:1000 dilution; Cell Signaling, #2775) overnight at 4°C followed by 1h of incubation with donkey-anti-rabbit or rat-anti-mouse detection antibody, respectively, at room temperature (1:2000 Jackson Laboratories, Bar Harbor, ME, USA). Signal was detected with the Biorad ChemiDoc™ XRS+System by enhanced chemiluminescence. Blots were subsequently quantified using ImageJ software (Ver 1.52R). HIF-1α, LC3B-I and LC3B-II levels were first corrected for loading by β-Actin, after which these levels were normalized to medium control or OxLDL respectively. Fold-changes are represented in bar graphs that were generated using GraphPad 6 software.

### Statistical analysis

Data were statistically analyzed by performing two-tailed nonpaired *t* tests using GraphPad Prism (version 6 for Windows; GraphPad Software, San Diego, CA) for comparing differences in ECAR% between control and oxLDL-treated KLM-1 cells. One-way ANOVA with multiple comparisons Tukey post-hoc test was used to compare differences of HIF-1α or LC3B-II between control and oxLDL treated KLM-1 cells pooled from 3 independent experiments. The results were visualized and expressed as the mean ±SEM and considered significant at *p* ≤ 0.05, * and ** indicate *p* ≤ 0.01 respectively.

## Results

### OxLDL increases glycolysis in KLM-1 cells

**Figure 1:**
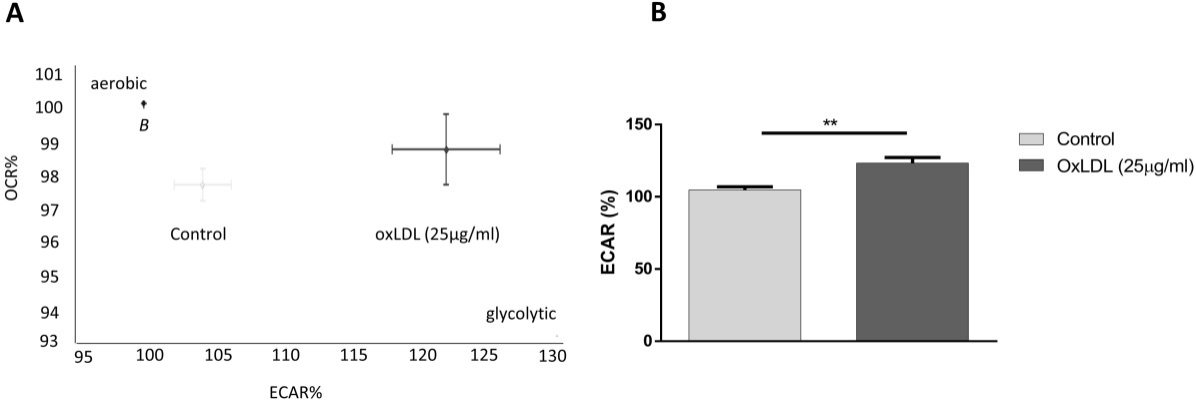
** OxLDL shifts energy balance to a glycolytic profile.** KLM-1 pancreatic cancer cells were titrated with oxLDL (25 µg/ml). (**A**) Representative energy metabolism diagram of basal OCR *vs.* ECAR. (**B**) Difference in ECAR% between control and oxLDL-treated cells. **p<0.01.

Oxygen consumption rate (OCR) is routinely used as an indicator of mitochondrial oxidative phosphorylation, whereas extracellular acidification rates (ECAR) reflect the state of lactate secretion into the medium, which is strongly linked to the levels of glycolysis in the cell. In order to investigate the effects of oxLDL on cellular energy balance in cancer cells, we titrated 25 µg/ml of oxLDL, under normoxic conditions, to the pancreatic tumor cell line KLM-1 and measured real-time basal changes in OCR and ECAR over the course of 60 min. Whereas no effect was observed on OCR rates **(supplemental Fig. 1)**, our data showed that incubation with oxLDL shifted the energy balance towards a more glycolytic phenotype, as shown by a 20% increase in ECAR compared to control cells (**Fig. 1A,B**).

### OxLDL stabilizes HIF-1α protein

**Figure 2:**
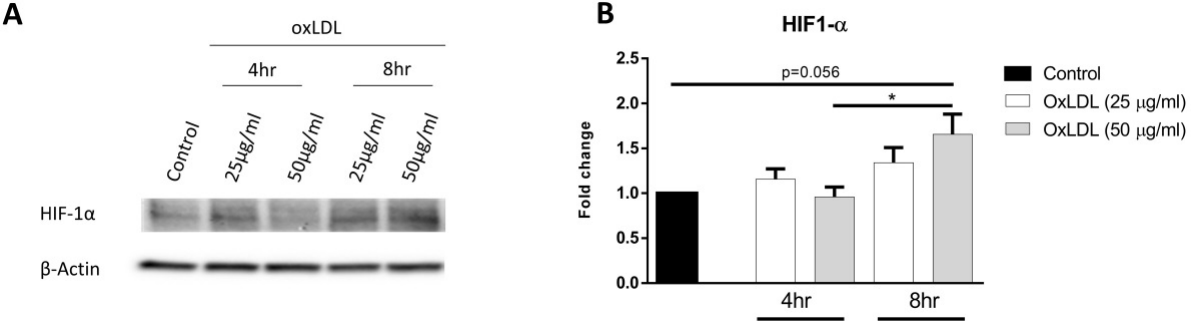
** OxLDL stabilizes HIF-1α protein levels.** KLM-1 cells were treated with oxLDL (25 or 50 µg/ml) for 4 hr or 8 hr. (**A**) Representative Western blot of HIF-1α protein levels, (**B**) Quantification of HIF-1α protein adjusted for β-Actin.

HIF-1α is a master regulator of glycolysis and high levels of the stabilized protein is maintained in cells with high glycolytic rates. In order to determine the effect of oxLDL on HIF-1α protein stabilization, KLM-1 cells were incubated in the absence or presence of different concentrations of oxLDL (25 or 50 µg/ml, respectively) for 4 hr or 8 hr. Our results demonstrated that protein levels of HIF-1α increased in response to 50 but not 25 µg/ml oxLDL treatment compared to control (p=0.056) (**Fig. 2A, B; Supplemental Fig. 2**). Furthermore, significant differences in HIF-1α protein were observed after incubation of 8 hr compared to 4hr, suggesting that the oxLDL-mediated increase in ECAR resulted from HIF-1α protein stabilization in a dose- and time-dependent manner.

### OxLDL increases autophagosomal marker LC3B-II

**Figure 3:**
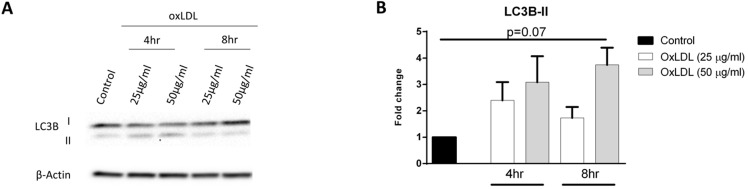
** OxLDL increases LC3B-II proteins levels.** KLM-1 cells were treated with oxLDL (25 or 50 µg/ml) for 4 hr or 8 hr. (**A**) Representative Western blot of LC3B-I and LC3B-II protein levels, (**B**) Quantification of LC3B-II protein adjusted for β-Actin.

In addition to its involvement in glycolysis, HIF-1α can also modulate autophagy in order to secure additional energy during metabolic stress responses such as hypoxia. It has been shown that the cytosolic protein LC3B-I is converted to autophagosomal marker LC3B-II during the fusion of endosomes with lysosomes, a process known as autophagosome formation 16. In order to assess whether autophagic activity is induced in response to oxLDL, we treated KLM-1 cells with or without oxLDL (25 and 50 µg/ml, respectively) over a time course of 4 hr and 8 hr. Subsequently, protein levels of LC3B-II were determined with Western blot (**Fig. 3A; Supplemental Fig. 2**) and quantitative data analysis showed that incubation with oxLDL resulted in a 4-fold increase of LC3B-II protein expression after 8 hr (p=0.07) (**Fig. 3B; Supplemental Fig. 2**).

### EO6 reverses oxLDL-induced metabolic changes

**Figure 4:**
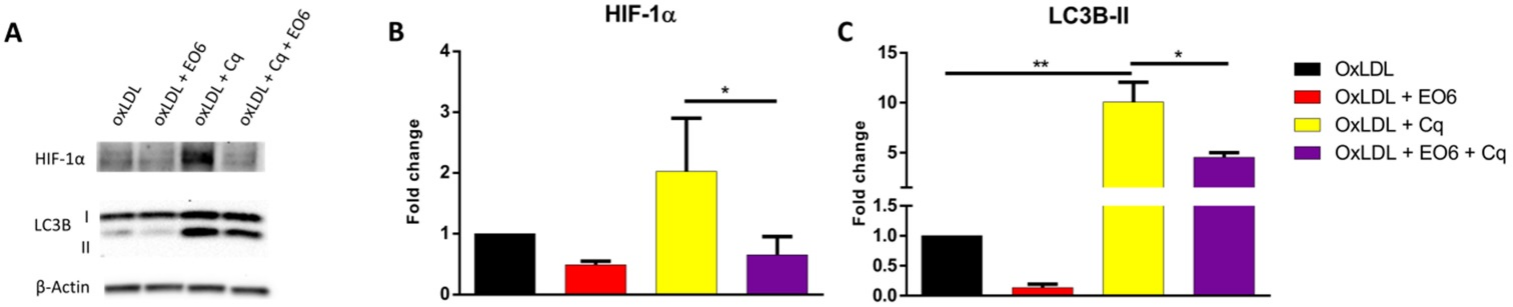
** EO6 reverses oxLDL-mediated effects on HIF-1α and LC3B-II.** KLM-1 cells were treated with oxLDL (50µg/ml) in the presence or absence of chloroquine (Cq; 20 µM) and EO6 antibodies against oxLDL (14 µg/ml) for 8 hr. (**A**) Representative Western blot of HIF-1α and LC3B-I and LC3B-II protein levels, (**B**) Quantification of HIF-1α and (**C**) LC3B-II protein adjusted for β-Actin.

Given the fact that natural IgM antibodies against oxLDL have been shown to ameliorate the adverse effects of oxLDL in several metabolic diseases, including NASH 17 and atherosclerosis 18, we assessed whether EO6 monoclonal antibodies against oxLDL were able to counteract the metabolic effects of oxLDL on HIF-1α and LC3B by treating KLM-1 cells for 8hr with oxLDL (50 µg/ml) in the presence or absence of EO6 (14 µg/ml). Additionally, cells were also treated with chloroquine (Cq; 20 µM) in order to ensure that any observed differences in LC3B-II were due to increased conversion of LC3B-I rather than differences in breakdown of LC3B-II. Chloroquine is a lysosomotropic weak base that diffuses into the lysosome, where it gets trapped, thereby changing the lysosomal pH and subsequently inhibiting autophagic degradation of proteins including LC3B-II 19. Our results showed that treatment with EO6 significantly reversed oxLDL-mediated increases in HIF-1α protein, in the presence of chloroquine (**Fig. 4A, B**). Similarly, treatment with EO6 also significantly reversed oxLDL-mediated increases in LC3B-II protein, in the presence of chloroquine (**Fig. 4A, C; Supplemental Fig. 2**).

## Discussion

Metabolic reprogramming, referred to as an important cellular adaptation mechanism, is unequivocally considered a hallmark of cancer 20. Many studies have focused on gaining better insight into the complex nature of cancer cell metabolism both *in-vitro* 21, 22 and *in-vivo* 23, and recently, the impact of abnormal lipid metabolism in modulating cancer pathophysiology has received increasing interest 24. In the current study, we provide novel insights into the role of oxLDL in regulating cancer cell metabolism.

Due to their high proliferation rate, cancer cells show a strong avidity for lipids and cholesterol, which they retrieve either from increased uptake of dietary lipids and/or cholesterol, or from enhanced lipogenesis and cholesterol synthesis 25, 26. One of the most prominent features associated with increased dietary lipids, and hence dyslipidemia, is low-density lipoprotein (LDL) oxidation. Oxidative modifications of LDL can occur as a result of elevated oxidative stress, a metabolic derangement known to be common in cancer and MetS-related diseases.

Previously, it has been shown that increased levels of oxLDL have detrimental effects on healthy metabolic tissues, including the cardiovascular system 27 and the liver 28. For instance, oxLDL impaired mitochondrial respiration in porcine aortic endothelial cells due to reduced activities of mitochondrial complexes 29, likely inducing a metabolic shift. Furthermore, overloading macrophages with oxLDL triggered a metabolic switch that resulted in high levels of HIF1-α 30, 31, which is a master regulator of metabolism that is constitutively expressed in cells. Normally, in the presence of oxygen, HIF-1α is destabilized and targeted for proteosomal degradation 32. However, under abnormal conditions such as hypoxia, which is a common feature of cancer, HIF-1α is being stabilized, thereby mediating a switch towards glycolytic metabolism 33. Alternatively, under normoxic conditions HIF-1α can also be stabilized in an oxygen-independent manner through the noncanonical HIF-1α activation pathway. Several factors such as oncometabolites (succinate/fumarate), heat shock proteins (Hsp90, Hsc70), regulators of tumor suppressor genes (MDM2 ligase) and changes in oxidative phosphorylation (OXPHOS) system are shown to affect HIF-1α stabilization. For example, tumorigenic factors are known to promote protein synthesis of HIF-1α via Akt pathway and Akt mediated synthesis of Hsp90 can further stabilize HIF-1α in an oxygen-independent fashion 34.

Given the fact that oxLDL stimulates the proliferation of a variety of cell types, including macrophages, endothelial and smooth muscle cells 35-37, oxLDL has been suggested to have pro-oncogenic properties. Indeed, in the context of cancer, it has been reported that increased accumulation of oxLDL, which is related to hyperlipidemia, is highly associated with the development of several types of cancer, including colon, breast and ovarian cancer 14, 38, 39. Nevertheless, the exact mechanism of oxLDL on cancer cell metabolism has not yet been fully understood.

Previous studies showed that metabolic derangements, such as hypoxia, can cause a remarkable upregulation of HIF-1α 40, triggering glucose uptake in mouse granulosa tumor cells 41. One way in which cancer cells become resilient or resistant, and thus enhance their capability to proliferate or metastasize, is by metabolically switching to glycolytic profiles and inducing cellular protective autophagy. Hence, several strategies, focused on reorienting cancer cell metabolism from glycolysis to oxidative phosphorylation, have demonstrated a beneficial decrease in cell survival, invasiveness and tumor growth. For instance, Poteet *et al.* showed that methylene blue was capable of increasing oxygen consumption while reducing lactate production, thereby reversing the glycolytic profile and decreasing proliferation of glioblastoma cell 42. Another study showed that resveratrol increased ATP production and thus the oxidative capacity, while decreasing glycolysis, as shown by a reduction in the pentose phosphate pathway in colon cancer cells 43. Moreover, they also demonstrated that resveratrol was able to modify the lipodomic profile of these cells, which is likely important for its metabolic effects on cancer energy metabolism. In the present study, we demonstrate for the first time that incubating KLM-1 pancreatic cancer cells with oxLDL, under normoxic conditions, leads to HIF-1α stabilization. These data are in line with others showing that oxLDL induced HIF-1α protein accumulation in human macrophages 30, 31. Furthermore, these data also support the observed increase in basal ECAR levels, pointing towards oxLDL as an inducer of a metabolic switch towards glycolysis in cancer cells. Given that cancer cells, even under non-hypoxic conditions, are capable of inducing HIF-1α and can actively shift their energy production from mitochondrial to glycolytic sources 44, our data suggest that treatment of oxLDL mimics a 'Warburg'-like effect in KLM-1 pancreatic cancer cells. This characteristic of oxLDL which promotes the switching to glycolytic metabolic profiles could potentially explain how oxLDL increases cancer cell survival and in so doing promote tumorigenesis & proliferation. In addition to this, another known method cancer cells utilize to increase survival and in so doing promote tumorigenesis & proliferation is by inducing cellular protective autophagy.

One of the most conserved physiological processes present in eukaryotes is autophagy, a catabolic pathway that is known to involve lysosomal degradation of organelles and cytoplasmic contents that are recycled for sustaining cellular energy requirements 45. It is known that under some circumstances, autophagy may be utilized as a cellular 'suicide' mechanism, thereby inducing cell death 46. Recent studies also discovered several protective functions for autophagy, including the regulation of intracellular lipid stores and a role in immunity, cell death and inflammation 47, 48. For instance, Kwanten *et al* demonstrated absence of fasting-induced steatosis, enhanced serum lipid profile, decreased glycemia using autophagy deficient mice. These findings underscored a role for the autophagy process in pathophysiology of diseases such as NAFLD/NASH among other liver diseases 49. In the context of MetS, we demonstrated that oxLDL disturbs lysosomal function 28 and induces autophagy 50, which was correlated with hepatic inflammation, whereas others found that elevated levels of oxLDL enabled the selection and survival of cancer cells due to its involvement in protective autophagy 51, suggesting a multifaceted role of oxLDL. Previous studies have also shown that metabolic derangements, such as hypoxia, can cause a substantial upregulation of HIF-1α 40, triggering cellular autophagy in mouse granulosa tumor cells 41. In line with these studies, we showed that oxLDL treatment resulted in an increase in the autophagosomal marker LC3B-II, which is a central protein in the regulation of autophagy 52. Hence, our data suggest that oxLDL stabilizes HIF-1α, thereby activating the autophagic pathway, as shown by the concomitant increase in LC3B-II protein. Based on other studies 53, 54, it is very likely that these changes are responsible for the oxLDL-induced metabolic switch towards glycolysis observed in KLM-1 pancreatic cancer cells, which is crucial for cell survival.

As previously discussed oxLDL has stabilizing effects on HIF-1α and activates the autophagic pathway, both of which are known to be involved in pro-oncogenic processes. In the current study, we also explored the ability of natural IgM antibodies against oxLDL (referred to as EO6) to prevent the changes in metabolic profile due to oxLDL in the context of cancer cell metabolism. Our results showed that EO6, by scavenging oxLDL, is capable of reducing the ability of oxLDL to stabilize HIF-1α and the subsequent increase in autophagy. Given that oxLDL is involved in many aspects of metabolic diseases, it has become a promising target in chronic inflammatory diseases, in which lipid metabolism is disturbed. Exercise training 55, 56, lowering dietary fat intake 57 as well as antioxidant 58, 59, statin 60 and fenofibrate therapies 61 are therapeutic strategies that have been involved in lowering oxLDL levels. Relevantly, these strategies have also been shown to reduce cancer risk 62 or exhibit anticancer properties 63-65, of which some therapeutic modalities have been already used in clinical trials. In the context of MetS, several studies have demonstrated that circulating levels of oxLDL can also be effectively targeted in a more direct manner without inducing side effects, as unfortunately often seen with adjuvant therapies 66. Recent evidence indicates that immunizing hyperlipidemic mice with *Streptococcus pneumoniae* is an effective way of triggering a natural immune response (*i.e.*, inducing an increase in monoclonal IgM autoantibodies) against oxLDL, thereby reducing atherosclerotic lesions 18. Similarly, in the context of NASH, we previously demonstrated that specific targeting of oxLDL by means of pneumococcal immunization also reduced hepatic inflammation 17, suggesting that the scavenging of oxLDL by anti-oxLDL autoantibodies might be a potent therapeutic strategy in cancer treatment. Obviously, chronic inflammation is also strongly associated with malignant diseases 67, 68 and tumor-associated macrophages are known to represent the major inflammatory cell population in tumors. Hence, several studies explored the effects of anti-inflammatory strategies, such as nonsteroidal anti-inflammatory drugs, and showed their ability to prevent early onset of cancer 69 and reduce the risk of cancer 70, thus underscoring the significance of inflammation during neoplastic progression.

## Conclusions

In the present study, we demonstrate for the first time that in addition to the known beneficial metabolic effects of the tumor microenvironment, oxLDL has a direct effect on pancreatic cancer cells by promoting a metabolic shift towards a more glycolytic phenotype. Furthermore, our data revealed that the addition of EO6 IgM antibodies against oxLDL were able to reverse these oxLDL-mediated metabolic effects. While there are several studies looking into the effects of oxLDL in other types of cancer, little is known about its effect in pancreatic cancer. As oxLDl is likely to also play a role in pancreatic cancer, future studies should therefore look into the effect of oxLDL in pancreatic cancer and evaluate the potential of targeting oxLDL as a novel therapeutic option in cancer.

## Supplementary Material

Supplementary figures.

## Abbreviations

Cq: chloroquine
ECAR: extracellular acidification rates
HIF-1α: hypoxia-inducible factor 1-alpha
LDL: low-density lipoprotein
MetS: metabolic syndrome
NASH: non-alcoholic steatohepatitis
oxLDL: oxidized low-density-lipoproteins
OCR: oxygen consumption rates
