# Supplementary material for: OxLDL as an Inducer of a Metabolic Shift in Cancer Cells
Source: J Cancer. 2021 Aug 3;12(19):5817–24. doi: 10.7150/jca.56307 (PMC8408103; doi:10.7150/jca.56307)
Supplement: Supplementary file 1 — Supplementary figures. [file jcav12p5817s1.pdf]

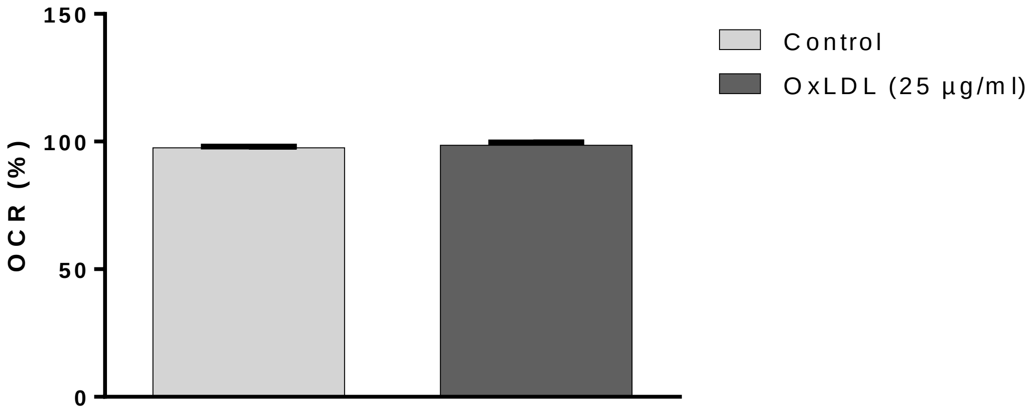

Supplemental Figure 1: OxLDL shifts energy balance to a glycolytic profile

KLM-1 pancreatic cancer cells were titrated with oxLDL (25µg/ml). Difference in OCR% between control and oxLDL-treated cells.

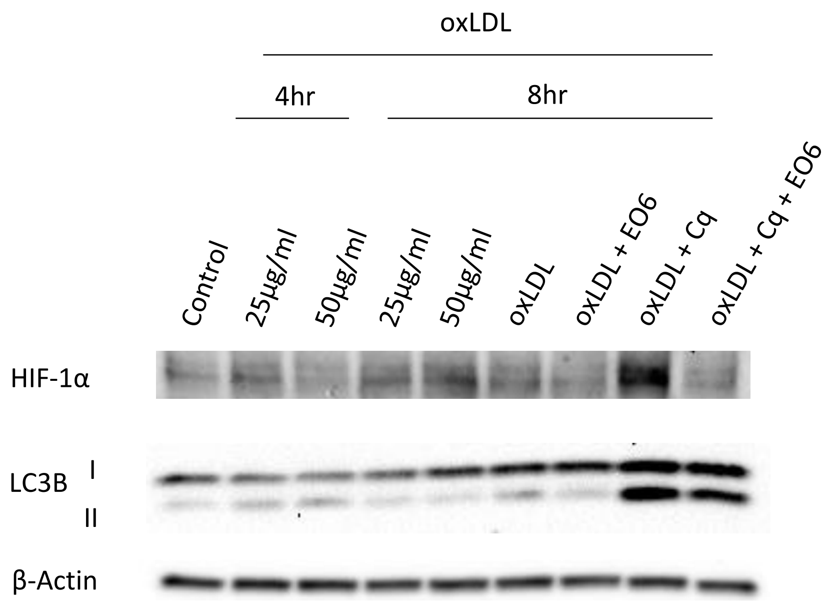

Supplemental Figure 2: OxLDL and EO6 affect HIF-1α and LC3B protein expression in KLM-1

A representative westernblot of HIF-1α and LC3B protein levels. KLM-1 cells were treated with oxLDL (25 or 50µg/ml) for 4hr or 8hr. Additionally, KLM-1 cells were treated with oxLDL (50µg/ml) or oxLDL (50µg/ml) + EO6 (14µg/ml) in the presence or absence of chloroquine (20µM) for 8hrs.
